# Effect of albumin and total protein concentration on plasma sodium measurements in the ICU

**DOI:** 10.1186/cc13623

**Published:** 2014-03-17

**Authors:** F Gennart, T Vanwynsberghe, M Nauwynck, M Bourgeois

**Affiliations:** 1 AZ Sint Jan Brugge, Bruges, Belgium

## Introduction

Direct ion-selective electrode without dilution is the most effective method for determination of the concentration of the ionized fraction of sodium [1]. We tested the hypothesis that the difference between indirect and direct sodium assays would be related to the plasma albumin concentration or the total protein concentration.

## Methods

A retrospective observational study was conducted in which plasma sodium concentrations, from 101 paired venous and arterial samples from patients admitted to the ICU, were respectively analyzed on the arterial blood gas (ABG) analyzers (direct ion-selective electrode) and the central laboratory auto-analyzers (indirect ion-selective electrode). A paired *t *test was performed comparing the central laboratory and ABG measurements. Correlation and regression analysis were performed between total protein concentration, albumin and the differences between the central laboratory and ABG assays for sodium.

## Results

The central laboratory sodium measurement was, on average, 1.46 mmol/l more than the ICU assay, limits of agreement 1.18 to 1.74 mmol/l greater, *P *< 0.001. Bland-Altman analysis of the central laboratory result minus the ICU sodium measurement had limits of agreement of 1.3 to -4.2 mmol/l. The correlation between the assay differences and total protein concentration and albumin were respectively *r *= 0.24 *(P *= 0.01) and *r *= 0.20 *(P *= 0.04). The difference in plasma sodium concentration between the assays increased as the plasma concentration albumin or total protein concentration decreased (respectively: *r*^2 ^= 0.04 and *r*^2 ^= 0.06).

## Conclusion

The difference between indirect and direct sodium assays was found to be statistically related to the plasma albumin concentration and the total protein concentration. Although the relationship was found to be weak, the total protein concentration should be monitored when measuring sodium by indirect ion-selective electrode.

## References

[B1] Burtis C Elsevier Tietz Textbook of Clinical Chemistry and Molecular Diagnostics 2006 983 1018

